# Does Individuals’ Perception of Wastewater Pollution Decrease Their Self-Rated Health? Evidence from China

**DOI:** 10.3390/ijerph19127291

**Published:** 2022-06-14

**Authors:** Shu Wang, Jipeng Pei, Kuo Zhang, Dawei Gong, Karlis Rokpelnis, Weicheng Yang, Xiao Yu

**Affiliations:** 1 School of Economics, Minzu University of China, 27 Zhongguancun South Avenue, Beijing 100081, China; wangshu@muc.edu.cn (S.W.); peijipeng1118@126.com (J.P.); 19300035@muc.edu.cn (K.Z.); vrl880723@163.com (W.Y.); 2 China Institute for Vitalizing Border Areas and Enriching the People, Minzu University of China, 27 Zhongguancun South Avenue, Beijing 100081, China; 3 School of Environment & Natural Resources, Renmin University of China, 59 Zhongguancun Avenue, Beijing 100872, China; gdw2019@ruc.edu.cn; 4 Council on International Educational Exchange, 600 Southborough Drive, Suite 104, South Portland, ME 04106, USA; karlisr@yahoo.com

**Keywords:** wastewater pollution, self-rated health, perception of environmental risk, industrial (agricultural/domestic) wastewater, China

## Abstract

Background: This study used original survey data to quantitatively investigate the associations between individuals’ perception of locally present wastewater pollution and their self-rated health. Methods: This research used the data from large-scale surveys covering all the 31 provinces and equivalent administrative units in mainland China and interviewed 6112 participants. The ordered logit method was employed to estimate the models. Results: The results indicated that individuals’ perceptions of local industrial and domestic wastewater pollution significantly decrease their self-rated health. If industrial wastewater pollution was reported, the possibility of the observers indicating lower levels of self-rated current health, comparing to the past year, and comparing with peers, all increased by 26% (*p* < 0.001), 23% (*p* = 0.005), and 18% (*p* = 0.006), respectively. Likewise, perceived domestic wastewater pollution led to the increase by 21% (*p* = 0.012), 17% (*p* = 0.034), and 33% (*p* = 0.000), respectively. Meanwhile, reported industrial wastewater pollution also has an obvious negative effect on individuals’ health performance, such as being more fatigued and upset. Conclusions: The survey clearly shows that Chinese individuals who are aware of water pollution in their living environment tend to experience more negative health outcomes, which adds additional urgency to improving wastewater treatment.

## 1. Introduction

Water pollution mainly includes industrial wastewater, domestic wastewater, and agricultural wastewater, all of which can cause various diseases and directly threaten public health and quality of life [1,2,3]. The relationship between water pollution and individuals’ actual health statuses has been well documented [4,5]. For example, water contaminated by toxic chemicals has been shown to cause acute and chronic poisoning through drinking water or food chain channels, such as Minamata disease caused by methylmercury poisoning and itai-itai disease caused by cadmium poisoning [6]. Long-term consumptions of water that contains carcinogenic chemicals, such as arsenic, chromium, and nickel, has been shown to cause cancer [7]. Furthermore, biological pollutants, such as human and livestock feces, pollute water sources and enter the human body through drinking water, leading to waterborne infectious diseases, such as dysentery, enteritis, and schistosomiasis [8].

At the same time, a number of studies have explored the direct relationship between water pollution and personal health from the perspective of social science, such as economics and sociology [9,10,11]. Some of the literature particularly focuses on the correlation of individual perception of water quality (pollution, taste, and color) and the related health status from a psychological angle [12,13,14]. Scholars have identified the concept of “perception of risk” to examine individuals’ judgements when they are asked to characterize and evaluate hazardous activities and technologies. Regardless of whether the judgements are right or wrong, the outcomes could help decision-makers to understand how non-professionals participants think about and respond to potential risk, and then take targeted actions [15]. Based on this, studies on the perception of environmental risk, such as the perception of natural hazards, garbage pollution, air pollution, light pollution, oil pollution, and water quality or pollution, can provide insights from the perspective of lay persons on their factually correct, or not, environmental perception [16,17,18,19]. Accordingly, it would be meaningful to assess the evidence of the connections between individual perception of wastewater pollution and health status using quantitative social sciences methods, thus contributing to the research area of perception of environmental risk.

Regarding personal health, self-rated health is one of the most commonly used indicators for personal health measurement. It has been widely used as the substitute variables of actual health status in social science research [20,21]. It requires individuals to score their overall health status from excellent to very poor (or from very poor to excellent) [22]. Self-rated health is a “subjective” indicator, which has been found to be a good predictor of mortality [23], future functional status [24], and outcome of treatment [25], indicating that self-rated health is closely related to medically confirmed health indicators [26]. Although self-rated health is simple as it is a one-dimensional-indicator, previous studies have confirmed that it is an inexpensive, useful, and efficient way to evaluate a person’s overall health status without medical examinations [27]. Meanwhile, the demographic, socioeconomic, and psychosocial determinants of self-rated health, such as gender, age, race, education, employment status, satisfaction, and income, have been confirmed and widely studied [28,29,30,31]. Following the existing literature, this research employed self-rated health as the measurement for individual health status in the study.

The United Nations Children’s Fund and the World Health Organization (2019) pointed out that about 2.2 billion people in the world do not have safe drinking water services, and 4.2 billion people are threatened by wastewater pollution due to a lack of safe management of water resources [32]. Due to the great destructive impact of wastewater pollution on public health and socio-economic development, many countries, including China, have taken to solving the problem of water pollution as an important part of government work, and put improving public environmental awareness and improved water treatment and management as the top priority [21,33,34,35]. For example, the Chinese central government has prepared the “Healthy China” strategy along with the “Healthy China Plan 2030”, which aims to ensure a good natural and social environment for healthy lives of the Chinese individuals [36]. Therefore, exploring the relationship between personal perception of environmental risk and individual self-rated health, such as the perception of wastewater pollution or air pollution, has practical and urgent current significance. However, the studies on the connection mainly focus on individuals’ perception of air pollution and their health status [37,38,39,40,41], while studies on personal perception towards wastewater pollution are rare, especially case studies from China. To supplement existing research, this study aims to investigate the connections between the Chinese individuals’ perception on wastewater pollution and their self-rated health. Further, this study poses a hypothesis that the perception of wastewater pollution in direct vicinity of an individual decreases the individual’s self-rated health status, which will be tested through empirical analysis.

## 2. Strategy

### 2.1. Data

This study employed the data from large-scale fieldwork started in January 2018 and focused on investigating the Chinese individuals’ environmental perception and attitudes. This research project complies with the Minzu University of China and the funding body’s research protocols and requirements. The surveys cover all the 31 provinces and equivalent administrative units in mainland China, including a substantial number of observations from all the 5 autonomous regions for ethnic minorities. By the end of 2021, the surveys had interviewed 6112 individuals. As China is heterogeneous between urban and rural sectors, the surveys intentionally cover both China’s urban and rural areas. It is believed that the large sample size is sufficient to meet research needs [42].

The surveys were conducted using face-to-face interviews, telephone interviews, as well as online questionnaires (after the start of the COVID-19 epidemic). The questionnaire covers respondents’ socioeconomic information, the individual’s views on environmental pollution and government’s environmental governance, the individual environmental perception, and the individual evaluation of health status and subjective wellbeing, which is provided in File S1.

**Figure 1:**
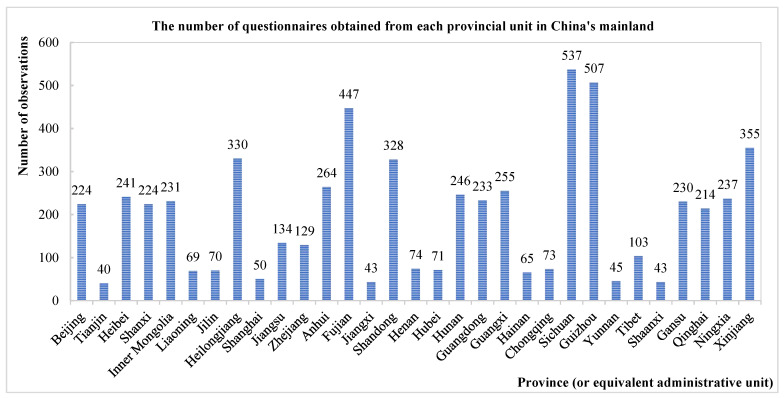
The number of questionnaires obtained in the surveys from each provincial-level unit of China’s mainland.

Time availability and budget were the major limitations that prevented the research from adopting a probabilistic sampling method (e.g., stratified random sampling) for the surveys. Meanwhile, as China is a large country with many ethnic groups, there are significant language, customs, as well as regional disparities, making it difficult to randomly carry out surveys that account for such differences. The affiliation with the Minzu University of China, the country’s leading institution for the research and training of ethnic minorities, the team members were able to apply their unique social connections to purposefully collect data in regions that are otherwise usually overlooked. As a result, the research employed a basic sociological method, “convenience sampling”, to access interviews with participants in the surveyed areas and obtain an insightful sample for analysis [42]. The surveys covered major cities, towns, and subordinate rural areas in all the provinces or equivalent administrative units. Accordingly, it is believed that the data could minimize sample selection bias at the provincial level, while the results gained from the surveys should be considered reliable and accurate. The number and regional distribution of the questionnaires is available in Table S1. The distribution of questionnaires in the surveys from each province (or equivalent administrative unit) of China’s mainland is shown in Figure 1.

As said above, the large-scale survey faced difficulties in several aspects, including limitation due to team members’ interpersonal relationship networks in each location. Therefore, the number of questionaries obtained from each provincial administrative unit varies. Nevertheless, many members of the research team, as well as students who participated in this project, are from China’s minority areas. This is of great value to gather relatively large amounts of first-hand data from five autonomous regions in China, including Xinjiang, Tibet, Inner Mongolia, Guangxi, and Ningxia, which are frequently excluded from other survey data. Meanwhile, the sample of 6112 individuals in this research is adequate to draw analytical conclusions. For one, most of China’s leading public survey data that is supported by the Chinese central government (e.g., the Chinese General Social Survey) mainly contain 10,000 or fewer observations, and seldom cover data from all the autonomous regions, especially Tibet. At the same time, many social science studies and published research on similar topics employ original survey data with samples that cover dozens, or hundreds of participants [13,14,19,36,38,39]. Therefore, it is believed that the observations that the surveys collected are of research value, and the sample could provide enough observations to support the analytical methods employed in the study.

### 2.2. Variables

#### 2.2.1. Dependent Variables

Individuals’ self-rated health is an important way to estimate the situation of public health, while certain subjective and objective factors, including environment, emotion, and cognition, may lead to the changes of health assessment levels [43]. This study used 3 discrete dependent variables to quantify the individual self-rated health as the dependent variables. Meanwhile, for exploring the specific impact of individuals’ perception concerned wastewater pollution on subjective health evaluation, the study also employed another 2 discrete variables that related to health as dependent variables. The reasons why 5 dependent variables were designed are as follows. On the one hand, the dependent variables contain several different measures of self-rated health, which enable the study to evaluate participants’ self-rated health comprehensively. On the other hand, the study would like to test the robustness of the model by employing different dependent variables, as well as different subsamples, to check the validity of the relationship between individual perception on wastewater pollution and self-rated health.

*Health-present*. The question for this discrete variable in the survey reads: “What do you think of your health status at present”, with value range from 1 to 5. More precisely, 1 meant “very healthy”, while 5 meant “very unhealthy”. The values 2, 3, and 4 meant “healthy”, “general”, and “unhealthy”, respectively.

*Health-past*. The question in the survey reads: “Compared to the last year, how do you evaluate your current health status”, with the values 1, 2, and 3, indicating “better than last year”, “almost the same”, and “worse than last year”, respectively.

*Health-peer*. The question in the survey reads: “Compared to your peers, how do you evaluate your current health status”. Similarly, the values 1, 2, and 3 meant “better than peers”, “almost the same”, and “worse than peers”, respectively.

*Fatigue*. It is believed that there is a direct link between water problems (e.g., quality, or contamination) and human health risk assessments, mental health, and well-being [44,45]. As typical mental health indicators [37,39,40], this research employs individual fatigue, as well as upset mood as another dependents. The question of measuring fatigue in the survey reads: “Are you fatigued with no reason”, values ranging was from 1 to 5, indicating “always”, “usually”, “sometimes”, “seldom”, and “never”, respectively.

*Upset*. Similar with the above, the study uses the question “Are you feeling upset and find it difficult to calm down”, to describe the typical health issue that the participants may face, with a value range from 1 (always) to 5 (never).

#### 2.2.2. Independent Variables

The individually reported presence of wastewater pollution is seen as the key independent variable, aiming to capture the connection between personal self-rated health and perception on the presence of wastewater pollution. The wastewater-related question on the questionnaire in the surveys reads: “What is the main type of wastewater pollution locally?”, and 5 answers were provided for this question, namely, “industrial wastewater”, “agricultural wastewater”, “domestic wastewater”, “other kinds of pollution”, and “do not know”, respectively. To be sure, a participant could report more than one answer for this question, which indicated that there were two or more types of local wastewater pollution that interviewees perceived. Accordingly, 3 dummy variables were design as the independents, which are listed below.

*Industrial*. If the participants perceived that industrial wastewater was the main kind of wastewater pollution locally, the value equaled to 1. Otherwise, the value equaled to 0. Similarly, this study also designed the dummy variables of “*Agricultural*” and “*Domestic*”, indicating that the value equaled to 1 if agricultural wastewater or domestic wastewater was perceived as the main kind of wastewater pollution.

**Figure 2:**
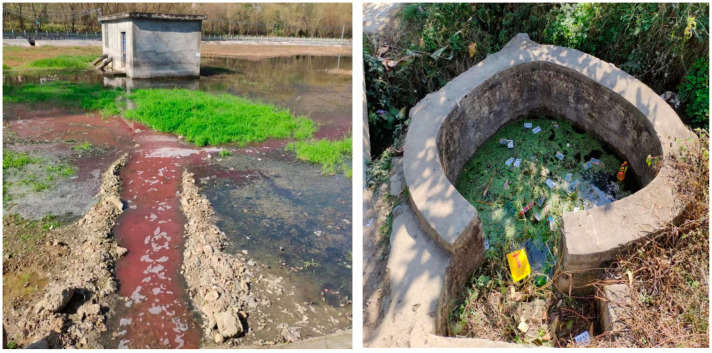
Perceived wastewater in the rural areas of Sichuan Province (taken in December 2018).

**Figure 3:**
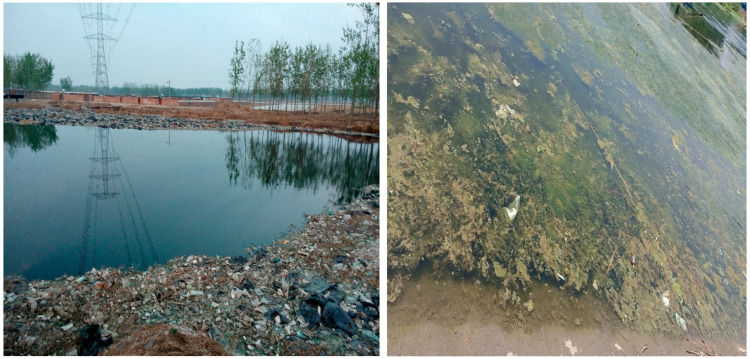
Water pollution in Hebei Province (taken in April 2018) and Fujian Province (taken in September 2019).

It is important to point out that according to former studies on perception of risk, as well as perception of environmental risk [15,16,17,18,19], with the participants being lay people and non-professionals, their judgements on the potential (environmental) risk may show great variability. However, the decision-makers could use the public’s either factually correct or biased perception to implement optional and targeted actions. During the surveys, the majority of participants reporting witnessing the wastewater discharge, and water contamination by garbage. They tended to judge water quality by smell, color, and visible pollutant. Therefore, as lay persons, they may not tell exactly right judgements in distinguishing the type of wastewater, as well as specific water quality. However, as has been argued in existing literature, the policymakers should see the outcomes as an important reference in environmental policy implantations [12,13,14,15]. Figure 2 and Figure 3 show the typically perceived wastewater and water pollution in the surveys.

In addition, several individual demographic and social characteristics were also considered as control variables, including gender, age, education status, and Hukou status, which might be the important factors in affecting personal self-rated health. More explanations are provided below.

*Gender*. It is a dummy variable; 0 and 1 indicated male and female, respectively.

*Age*. The questionnaire designed interviewee’s ages into groups, namely, under 30 years old, between 31–50 years old, and above 50 years old, and assigned values of 1, 2, and 3 respectively to the options of the 3 groups. However, this study needs to transfer a multivariate discrete variable into several dummy variables as independents. Accordingly, 2 dummy variables were designed to cover the participants’ age. They are: 31–50 years old and other age range (value as 1 and 0, respectively); and over 50 years old and other age range (value as 1 and 0, respectively).

*Education*. The options for the participant’s educational level were 1, 2, 3, 4, and 5, which represented primary school or below, middle school, high school, college or university, and a master’s degree or above, respectively. Similar with handling the variable of individual age, 4 dummy variables were employed to cover all the respondents’ educational levels. They are: middle school and other educational levels (value as 1 and 0, respectively); high school and other educational levels (value as 1 and 0, respectively); university (or college) and other educational levels (value as 1 and 0, respectively); and master’s degree (or higher) and other educational levels (value as 1 and 0, respectively).

*Hukou*. A Hukou is a legal document that records Chinese individuals’ basic information and allows the holder of a Hukou of a certain place and type (there are two types of Hukou: agricultural and non-agricultural) to access the corresponding social resources, such as educational and public medical insurance. Existing studies tend to follow the legally prescribed categories and distinguish urban and rural individuals by dividing their Hukou status [20,46]. Following the precedent, this study also divided the urban and rural participants by Hukou type. Therefore, Hukou was designed as a dummy variable, while 1 and 0 indicated Non-agricultural and Agricultural Hukou.

*Income*. Divided by the participants’ Hukou status and survey year, the study defined the interviewee’s income as the per capita disposable income of urban residents (Non-agricultural Hukou) or the per capita net income of rural residents (Agricultural Hukou) in his/her province (or equivalent administrative unit) in 2020, which is the newest public data from the “China Statistical Yearbook 2021”.

### 2.3. Methodology

In this study, all the 3 dependent variables are discrete variables, so the most common used models are logit or probit models, which report similar estimating results, but the error term is normally distributed, and logistic distributed, respectively. Accordingly, both ordered logit and ordered probit models can be applied under the situation of ordered responses. However, scholars are more accustomed to employ the method of ordered logit to estimate the model in this case [20]. Accordingly, ordered logit method was employed to estimate the model.

More specifically, the research estimates the following models:

$$Y_{i}^{*}=\alpha_{0}+X_{i}\delta +C_{i}\theta +\varepsilon_{i}$$

In the model, Y* is the latent variable of *Y*, while *Y* indicates dependent variables, including Health-present, Health-past, Health-peer, Fatigue, and Upset; $\alpha_{0}$ is an intercept term; $X_{i}$ is a vector of key independent variables that relate to individual perception on wastewater pollution; δ is the coefficient vector of X; $C_{i}$ is a vector of control variables; θ is the coefficient vector of C; and $\varepsilon_{i}$ is the residual term. Lastly, the study uses the statistical and econometric software STATA (version 16.0, StataCorp LLC, College Station, TX, USA) for empirical analysis. In addition, robustness tests (including substitution of dependent variables, and regression on subsamples), as well as significance value (*p*-value) are also used.

## 3. Results

Table 1 shows the general description of the sample. On the one hand, over 60% of the participants evaluated themselves as healthy or very healthy at present, while over half of the individuals believed their health status was almost the same as in the past, or comparable with peers. However, the majority of the interviewees reported that they seldom or sometimes experience fatigue and are upset. On the other hand, wastewater pollution was frequently reported by the participants. Around 40% of individuals perceived industrial wastewater as the main type of wastewater pollution locally, while 26.06% and 62.95% perceived agricultural and domestic wastewater as the main type, respectively.

As the study focused on the relationship between individuals’ perception of wastewater (industrial, agricultural, and domestic wastewater) pollution and their self-rated health, the distribution of health variables among the perception of different types of wastewater pollution was listed in Table 2. On reporting the present health status, individuals tended to evaluate themselves “unhealthy” if they perceived any type of wastewater pollution locally, no matter what type of wastewater. Similarly, interviewees were likely to evaluate their health status as “worse than the past year” or “worse than the peers” if they perceived industrial wastewater pollution, as well as domestic wastewater pollution locally. On the specific performance of individuals’ health evaluations, individuals tended to report that they felt fatigue if they also perceived wastewater pollution in their vicinity. However, it is difficult to speculate about the relationship between the frequency of individuals’ negative feelings and measurable wastewater pollution. A further empirical study would be needed.

Basing on the analysis above, the study took all the control variables into consideration to confirm whether control variables explain additional information on individual self-rated health except the influence of his or her perception of wastewater pollution. Meanwhile, this research also would like to provide a robustness test by comparing the results of all the models. Accordingly, Table 3 presents the results of Model 1 to Model 3 by using the ordered logit method, which shows the influence factors of Chinese individuals’ self-rated health. In addition, the OR (odds ratio) value for reporting each result of the models was also listed, which is convenient for analyzing the empirical results.

The results of the ordered logit model in Table 3 showed that the individual perception of wastewater pollution, along with some individual characteristics, significantly affects the respondents’ self-rated health. First, the participants who perceived industrial wastewater pollution locally tended to mark lower levels of self-rated health (at an odds ratio of 1.26). Specifically, if the independent variable “*Industrial*” leveled up from 0 to 1, that is, if a respondent perceived industrial wastewater pollution locally (*Industrial* = 1), the possibility of the individual’s self-rated present health (*Health-present*) stepping up a notch or more notches (worse self-rated health) increased 0.26 times (*p* < 0.001). In another word, the individual perception of industrial wastewater pollution led to a 26% increase in local individuals’ rating lower levels on the present health status. Similarly, the individual perception of industrial wastewater pollution also caused a 23% (*p* = 0.005) and an 18% (*p* = 0.006) rise in interviewees’ responding to lower levels on the health situation of comparing to the past year (*Health-past*) and with peers (*Health-peers*), respectively. Second, individuals were more likely to mark lower levels of self-rated health if domestic wastewater pollution was perceived. More precisely, if an individual perceived domestic wastewater pollution locally, the possibility of him or her evaluating a worse self-rated health status currently, compared to the past year and compared with peers, increased by 21% (*p* = 0.012), 17% (*p* = 0.034), and 33% (*p* = 0.000), respectively (at an odds ratio of 1.21, 1.17, and 1.33, respectively). Third, from the results of Model 1, Model 2, and Model 3, none of the estimated coefficients of the independent “*Agricultural*” was significant (*p* > 0.1). Accordingly, there is no evidence to prove that agricultural wastewater pollution reports would influence individuals’ self-rated health, for reasons that will be explained in the end of this section.

In addition, as mentioned above, for the investigation of the specific effects of perceived wastewater pollution on individuals’ health evaluations, the study also employed two other discrete variables that relate to health as dependent variables, namely, “*Fatigue*” and “*Upset*”. Accordingly, Model 4 and Model 5 also need to be estimated. Following the operations on Model 1 to Model 3, the regression results are listed in Table 4.

From Table 4, the empirical results of Model 4 and Model 5 are similar with the former three models. It is believed that if industrial wastewater pollution was perceived, the possibility of the participants evaluating a better health performance, namely feeling a lower frequency of fatigue and discomfort, decreased by 22% (*p* = 0.001) and 18% (*p* = 0.001), respectively (at an odds ratio of 0.78, and 0.82, respectively). However, individuals’ agricultural and domestic wastewater pollution reports lacked explaining power for the changes in the specific performance of their health status (*p* > 0.1). In addition, Model 1 to 5 provide the similar conclusions under the estimations by employing different dependent variables and subsamples, which indicates that the empirical results are robust and reliable.

It is widely reported that fertilizers are overused in China’s agriculture, leading to inefficient fertilizer utilization and nutrients loss. China’s agricultural wastewater consists mainly of irrigation water and run-off polluted by the nutrients of fertilizers [47]. The nutrients unabsorbed by plants (e.g., nitrogen and phosphorus) leak into the water system and cause agricultural wastewater pollution, which is difficult to perceive by individuals [48]. As mentioned above, participants judged water quality by witness of wastewater discharge, water smell, color, as well as the visible pollutants. Therefore, it is believed that as lay persons, the survey participants tended to perceive industrial and domestic wastewater more easily, but found it hard to perceive agricultural wastewater, which may lead to the bias of perception on agricultural wastewater [49]. In addition, in China’s case, individuals have limited direct exposure (e.g., drinking, washing, or usage in the daily life) to agricultural wastewater [50], so the perception of agricultural wastewater may not be the reason that participants reported lower levels on self-rated health.

Summing up the above, the research tested the hypothesis of the study through empirical analysis and found that the participants’ perception of wastewater pollution significantly decreased their self-rated health, which indicates that the hypothesis of this study is correct. More exactly, if individuals perceived that industrial and domestic wastewater pollution existed locally, they tended to report a lower level on their self-rated health. Meanwhile, perceived industrial wastewater pollution also has a negative effect on participant’s health performances, while they may have a higher possibility to feel fatigued and upset. Therefore, in China’s case, it is believed that individual perception of environmental risk, such as wastewater pollution, as well as water quality, does have significant impacts on his/her subjective evaluation of health status.

## 4. Discussion

Using the data from the large-scale surveys that covered all the 31 provinces and equivalent administrative units of mainland China, the relationship between perceived wastewater pollution and individual self-rated health was studied. It is found that reports on certain types of wastewater pollution, especially industrial and domestic wastewater, decreased individuals’ level of self-rated health. The study also discussed the potential impact that perceived wastewater pollution can have on the individual self-rated health. In this section, the research will discuss the connections of perceived wastewater pollution and self-rated health from a global perspective and raise some policy action.

Earlier studies believe that the perception of risk is central to many health behavior theories [51,52]. Particularly, environmental risk perceptions are often anchored in individual and familial experiences with health problems such as cancer and asthma [53]. In fact, certain studies find that there is a strong connection between the perception of environmental risk and mental health, while greater perceived potential environmental pollution, even biased perceived environmental indicators, are associated with poorer personal mental health [51,54], as well as high levels of distress (e.g., worry, annoyance, and intolerance) and disease symptoms [55]. Accordingly, as an important subjective environmental measure, scholars insist that the perception of environmental risk significantly affects individual subjective health, namely, self-rated health [56,57,58].

As expected, this study found that individuals’ perceptions of wastewater pollution, which is a major indicator of environmental risk perceptions, significantly decrease their self-rated health. The empirical results conform to the prevailing view on the connections of environmental risk perception and subjective health mentioned above. Regarding the underlying reasons of why wastewater pollution decreases one’s self-rated health, it has been shown and is often discussed in public forums that wastewater contains high concentrations of persistent organic pollutants, poisonous heavy metal, antibiotics, microplastics, and endocrine disruptors, which makes individuals suffer from a variety of diseases and threatens people accessing clean water. As a whole, this causes serious problems of food safety, resulting in individuals’ poor expectations on health [59,60]. At the same time, wastewater pollution may also have indirect influences on increasing individual purchase on medical services, reducing personal work efficiency and potential income, as well as decreasing one’s subjective wellbeing [61,62], which is harmful to the improvement of individual health level [63]. In fact, the analysis and results of this study come from the Chinese case and data, but it is believed that this study could be linked on a global prospective, because other studies on observing different countries provide similar findings, indicating that the perception of water pollution, contamination, and quality significantly influences personal health-related factors. Accordingly, a comparative table (Table 5) is provided below, which summarizes a selection of typical studies of this or similar topics from other countries.

In summary, based on the global perspective, it is common that individuals’ perceptions of environmental risk, such as perceived wastewater, as well as its resulting pollution, has strong connections on public health. Therefore, it is believed that the suggestions below are applicable to China.

On the one hand, China has been experiencing an economic boom during the past decades, with increased attention paid to environmental governance. As Table 6 shows, there is no obviously downward trend of total amount of wastewater discharge. However, compared with the other countries/regions, China enforces relatively high standards on the permissible limit of wastewater discharge, while the controls of the majority of key pollutants shows an improving trend. Therefore, in order to achieve better environmental management outcomes, as well as avoid the negative impact of wastewater pollution on public health, it is of importance for the Chinese government to control the amount of wastewater discharge and continue to strengthen the treatment on wastewater.

On the other hand, on the supply side of environmental policies, the Chinese government needs to meet public requirements by referencing their perception of environmental risk, which could reflect individuals’ urgent demands and expectations on the environment. Responding to the individuals’ thoughts and hopes for water quality, focusing on providing high-quality water, improving water treatment facilities should be a major concern. Meanwhile, as individuals may misperceive wastewater, water quality, as well as water pollution, the local governmental should enhance environmental education, data availability, and public accountability on water quality and wastewater treatment. Accordingly, relevant environmental policies and actions aiming at improving individuals’ perception of environmental risk, so as to increasing public health levels, should be considered as both an indicator of existing problems and priorities to be addressed.

## 5. Strengths and Limitations

The major strengths of this study are as follows. Firstly, this research employed data from large-scale surveys from 2018 to the present into the research, covering all the 31 provinces and equivalent administrative units, while 6112 individuals participated, which is brand new and first-hand data. Therefore, the analysis and conclusions are unique and insightful. Secondly, it is believed that this study provides certain theoretical contributions. In recent years, scholars have focused on the direct relationship between subjective environmental factors and individuals’ actual or objective health conditions, neglecting to pay attention to the connections between individual perception on environmental risk and personal health status. Accordingly, this study is a helpful supplement to the existing studies of the individual perception of environmental risk, such as wastewater and water contamination and quality, on the social science perspective. Thirdly, it is believed that the findings of this research have good applicability. This study provides evidence that the Chinese individuals’ perceptions of wastewater pollution significantly decrease their self-rated health. These conclusions are in line with the observed situations of many other countries in different continents and provide an important contribution to the global view of this matter. However, this study faces certain limitations. On the one hand, although this study tests the robustness of the models, and controls most unobserved factors, COVID-19 may still more or less affect participants’ self-rated health, which needs further discussion in the future research. On the other hand, the study only provides evidence of the relationship between the individual perception of wastewater pollution and subjective health status, while the corresponding data on the factual impact on participants’ health cannot be provided. In fact, a mixed study through medical, microbiological, and chemical analyses to complement the survey of perceptions would be a major enhancement and is a promising research path forward.

## 6. Conclusions

By employing the data from the large-scale surveys, which cover all the 31 provinces and equivalent administrative units in mainland China and 6112 participants, the connections between individuals’ perceptions of wastewater pollution and their self-rated health were studied. The research divided the wastewater into three categories, namely, industrial wastewater, agricultural wastewater, and domestic wastewater, and it is found in the study that the participants’ perception of industrial and domestic wastewater pollution significantly decreases their self-rated health. Meanwhile, perceived industrial wastewater pollution also has an obvious negative effect on individual health performance, such as feeling fatigued and unwell. Accordingly, relevant environmental policies aiming at reducing individuals’ environmental risks, as well as increasing public health levels, should be considered as priorities by the Chinese government. However, the negative effects of COVID-19 on individual self-rated health should be discussed deeply in the future similar studies. Hopefully, this topic will also attract attentions from the perspective of interdisciplinary research.

## Figures and Tables

**Table 1:** Characteristics of the study sample.

Variables	Category	N	Ratio (%)
Health-present	Very Healthy	887	14.51
	Healthy	2818	46.10
	General	1446	23.65
	Unhealthy	472	7.72
	Very unhealthy	95	1.55
	Missing value	395	6.46
Health-past	Better	1050	17.18
	Same	3389	55.44
	Worse	1273	20.82
	Missing value	401	6.56
Health-peers	Better	1178	19.27
	Same	3812	62.36
	Worse	727	11.89
	Missing value	396	6.48
Fatigue	Always	189	3.09
	Usually	493	8.06
	Sometimes	1069	17.49
	Seldom	983	16.08
	Never	331	5.41
	Missing value	3048	49.86
Upset	Always	124	2.03
	Usually	304	4.97
	Sometimes	1029	16.83
	Seldom	1131	18.50
	Never	476	7.79
	Missing value	3049	49.88
Industrial	No	3131	51.22
	Yes	2434	39.82
	Missing value	548	8.96
Agricultural	No	3972	64.98
	Yes	1593	26.06
	Missing value	548	8.96
Domestic	No	1717	28.09
	Yes	3848	62.95
	Missing value	548	8.96
Gender	Male	2818	46.10
	Female	2990	48.91
	Missing value	305	4.99
Hukou	Agricultural Hukou	2655	43.43
	Non-agricultural Hukou	2802	45.84
	Missing value	656	10.73
Age	0–30	3876	63.41
	31–50	1561	25.54
	≥51	389	6.36
	Missing value	0	0
Education	Primary school and lower	242	3.96
	Middle school	925	15.13
	High school	1434	23.46
	University (or college)	2894	47.34
	Master’s degree (or higher)	348	5.69
	Missing value	0	0

Note: “N” indicates the number of observations. The sample sets missing values including two conditions: interviewees chose “don’t know” for answers or did not answer at all (including the situation of individuals refusing to answer). Exactly, the question “What is the main type of wastewater pollution locally” in the questionnaire contains the option of “don’t know”, while 431 and 127 participants chose “don’t know” and did not answer, respectively (seen as a total of 548 missing values). Meanwhile, the other questions concerning the relevant variables in the questionnaire do not contain the option of “don’t know”, so the missing values came from the situation that individuals did not answer the question (or refused to answer). In addition, the response number of the variables “*Fatigue*”, and “*Upset*” is 3065 and 3064, which accounts for 50.15% and 50.13% of the total participants, respectively. It should be noted that the initial version of the questionnaire did not include the two questions mentioned above; the research project added these questions in the end of 2019 as they were identified as important enhancements to the survey.

**Table 2:** The response distribution between health-related variables and wastewater (%).

Health-present		Very Healthy	Healthy	General	Unhealthy	Very Unhealthy
		15.24	49.34	25.40	8.30	1.71
Industrial	Yes	14.06	48.00	26.80	9.07	2.06
	No	16.16	50.38	24.30	7.70	1.44
Agricultural	Yes	15.69	47.39	25.27	9.14	2.52
	No	15.07	50.13	25.46	7.96	1.39
Domestic	Yes	14.61	50.14	25.29	8.36	1.59
	No	16.67	47.54	25.65	8.16	2.00
Health-past		Better	Same	Worse		
		18.33	59.22	22.45		
Industrial	Yes	17.41	57.01	25.58		
	No	19.05	60.94	20.01		
Agricultural	Yes	19.50	59.56	20.95		
	No	17.86	59.08	23.05		
Domestic	Yes	18.02	59.17	22.82		
	No	19.04	59.34	21.62		
Health-peers		Better	Same	Worse		
		20.43	66.88	12.69		
Industrial	Yes	20.54	65.68	13.78		
	No	20.34	67.82	11.84		
Agricultural	Yes	21.06	66.14	12.80		
	No	20.18	67.18	12.64		
Domestic	Yes	19.74	66.77	13.49		
	No	21.99	67.14	10.88		
Fatigue		Always	Usually	Sometimes	Seldom	Never
		6.05	16.07	35.12	32.04	10.72
Industrial	Yes	7.09	16.19	36.05	31.04	9.63
	No	4.94	15.95	34.12	33.12	11.87
Agricultural	Yes	5.52	17.17	35.41	31.45	10.44
	No	6.26	15.63	35.00	32.28	10.83
Domestic	Yes	5.55	16.40	34.34	33.21	10.49
	No	7.07	15.40	36.71	29.64	11.18
Upset		Always	Usually	Sometimes	Seldom	Never
		3.91	9.79	33.71	37.21	15.39
Industrial	Yes	4.42	9.58	35.10	35.23	15.67
	No	3.36	10.01	32.24	39.31	15.08
Agricultural	Yes	3.96	11.28	33.49	36.25	15.01
	No	3.89	9.18	33.80	37.59	15.54
Domestic	Yes	3.19	9.62	33.38	39.51	14.30
	No	5.38	10.13	34.39	32.49	17.62

Note: The horizontal sum of data in each row equals to 100%.

**Table 3:** The influence factors of Chinese individuals’ self-rated health.

Variable	Category	Model 1 Health-Present		Model 2 Health-Past		Model 3 Health-Peers	
		OR (95% CI)	*p*	OR (95% CI)	*p*	OR (95% CI)	*p*
Industrial (wastewater)		1.26 (1.12–1.42)	<0.001	1.23 (1.06–1.42)	0.005	1.18 (1.05–1.32)	0.006
Agricultural (wastewater)		1.08 (0.93–1.26)	0.334	0.92 (0.81–1.04)	0.165	0.97 (0.81–1.17)	0.783
Domestic (wastewater)		1.21 (1.04–1.41)	0.012	1.17 (1.01–1.35)	0.034	1.33 (1.14–1.55)	0.000
Gender	Female	0.98 (0.85–1.14)	0.816	1.27 (1.10–1.48)	0.001	1.28 (1.09–1.50)	0.002
Hukou	Non-Agricultural	1.61 (0.63–4.11)	0.319	1.18 (0.41–3.39)	0.761	2.89 (1.16–7.20)	0.022
Age	31–50	1.40 (1.18–1.68)	<0.001	1.22 (1.00–1.49)	0.056	1.14 (0.91–1.43)	0.245
	≥51	1.35 (1.06–1.72)	0.015	1.12 (0.96–1.32)	0.154	0.87 (0.63–1.19)	0.371
Education	Middle school	0.85 (0.71–1.01)	0.067	0.94 (0.68–1.31)	0.719	0.85 (0.67–1.08)	0.174
	High school	0.77 (0.61–0.97)	0.025	0.88 (0.59–1.31)	0.522	0.70 (0.54–0.91)	0.007
	University	0.76 (0.61–0.97)	0.027	1.08 (0.73–1.60)	0.700	0.66 (0.51–0.86)	0.002
	Master’s and higher	0.68 (0.52–0.88)	0.004	1.08 (0.67–1.75)	0.747	0.62 (0.45–0.85)	0.003
Income	Local	0.70 (0.25–1.93)	0.489	0.79 (0.25–2.47)	0.682	0.30 (0.11–0.84)	0.003
FE	Province	Yes		Yes		Yes	
Cluster	Province	Yes		Yes		Yes	

Note: “OR” indicates odds ratio, “CI” indicates confidence interval, “*p*” indicates *p*-value, which shows the significance level, “FE” indicates fixed effect (the same below). In addition, “Local” indicates a participant’s location (province). Here the study controlled the economic development status, and residents’ income level in a participant’s province (or equivalent administrative unit) by employing the independent variable of “Income”.

**Table 4:** The specific effects of wastewater pollution on individuals’ health evaluations.

Variable	Category	Model 4 Fatigue		Model 5 Upset	
		OR (95% CI)	*p*	OR (95% CI)	*p*
Industrial (wastewater)		0.78 (0.68–0.90)	0.001	0.82 (0.73–0.93)	0.001
Agricultural (wastewater)		0.93 (0.78–1.11)	0.417	0.97 (0.82–1.15)	0.730
Domestic (wastewater)		1.08 (0.93–1.24)	0.306	1.09 (0.91–1.31)	0.349
Gender	Female	1.08 (0.93–1.25)	0.306	1.15 (0.97–1.35)	0.103
Hukou	Non-Agricultural	1.25 (0.35–4.44)	0.731	1.19 (0.36–3.88)	0.777
Age	31–50	1.02 (0.81–1.29)	0.845	1.24 (1.01–1.53)	0.044
	≥51	2.02 (1.43–2.83)	<0.001	1.66 (1.13–2.43)	0.010
Education	Middle school	0.81 (0.58–1.13)	0.219	0.86 (0.59–1.25)	0.415
	High school	0.90 (0.59–1.37)	0.631	0.84 (0.49–1.44)	0.538
	University	1.18 (0.80–1.75)	0.399	1.10 (0.68–1.78)	0.695
	Master’s and higher	1.29 (0.82–2.02)	0.271	1.19 (0.73–1.96)	0.483
Income	Local	0.65 (0.15–2.87)	0.572	0.76 (0.20–2.91)	0.691
FE	Province	Yes		Yes	
Cluster	Province	Yes		Yes	

**Table 5:** Similar studies in other countries under a comparative and global perspective.

Continent	Country	Authors	Main Point
America	USA	Covert et al. (2020) [64]	Paricapants’ concerns with water quality has important role in acting on their environmental health risk.
	USA	Merkel et al. (2012) [65]	Due to the pediatric health concerns, parents tended to worry about potential contamination of tap water.
	Canada	Ford et al. (2019) [66]	Households contradicted their perception and consumed water perceived as unsafe, while integration of risk perception lowered the adult incremental lifetime cancer risk.
	Brazil	Caputo (2022) [67]	There is a wide range of subjective perceptions and beliefs about drinking water quality and its impact on health that can diversely affect human behavior.
Africa	Kenya	Gevera et al. (2022) [14]	The increased health risks associated with high salinity and high F− in drinking water in Makueni County are poorly understood by most residents.
	Algeria	Benameur et al. (2021) [68]	The public knowledge about water pollution-related issues remains low, which affects policy maker’s actions for water contamination prevention and public health protection.
	Ghana	Kangmennaang et al. (2020) [69]	Participants not only hold various perceptions regarding the safety and quality of vended water but expressed emotional distresses such as discomfort, and anxiety.
Europe	Portugal	De França Doria et al. (2005) [70]	Perceived water quality, which is a risk indicator, seems to be mainly a result of external information, past health problems, and water colour.
Asia	Pakistan	Ahmed and Shafique (2019) [51]	There is a strong connection beween the risk perception of households regarding water pollution in Pakistan and its potential effect on human health.

**Table 6:** China’s wastewater discharge, key pollutants, and permissible limit comparison.

Year	Total Amount of Discharge (2011–2020)						
	Wastewater	COD	NH_3_-N	TN	TP	Petroleum	Volatile Phenol
	(Unit: 10,000 tons)	(Unit: 10,000 tons)	(Unit: 10,000 tons)	(Unit: 10,000 tons)	(Unit: 10,000 tons)	(Unit: tons)	(Unit: tons)
2011	6,591,922	2499.9	260.4	447.1	55.4	21,012.1	2430.6
2012	6,847,612	2423.7	253.6	451.4	48.9	17,493.9	1501.3
2013	6,954,433	2352.7	245.7	448.1	48.7	18,385.3	1277.3
2014	7,161,751	2294.6	238.5	456.1	53.5	16,203.6	1378.4
2015	7,353,227	2223.5	229.9	461.3	54.7	15,192.0	988.2
2016	7,110,954	658.1	56.8	123.6	9.0	11,599.4	272.1
2017	6,996,610	608.9	50.9	120.3	7.0	7639.3	244.1
2018	——	584.2	49.4	120.2	6.4	7157.7	174.5
2019	——	567.1	46.3	117.7	5.9	6293.0	147.1
2020	——	2564.8	98.4	322.3	33.7	3734.0	59.8
**Country/Region**		**Maximum Permissible Limit**					
		**COD**	**NH_3_-N**	**TN**	**TP**	**Petroleum**	**Volatile Phenol**
		**(Unit: mg/L)**	**(Unit: mg/L)**	**(Unit: mg/L)**	**(Unit: mg/L)**	**(Unit: mg/L)**	**(Unit: mg/L)**
China		120	25 (30)	20	5	15	0.5
USA		——	——	8	1	——	——
European Union		125	——	15	2	——	——
Japan		160 (120)	——	120 (60)	16 (8)	30	5
Singapore		100	——	——	——	10	0.2
Malaysia		200	50	——	10	10	——

Data source: (1) China’s wastewater discharge and key pollutants: Annual Report of China Ecological and Environmental Statistics 2011–2020 (see https://www.mee.gov.cn/hjzl/ (accessed on 2 May 2022)). The total amount of wastewater discharge in 2018, 2019, and 2020 are no longer reported in the annual reports. (2) Permissible limit of wastewater discharge: China: Cities Sewage Treatment Plant Pollutant Discharged Standard (GB18918-2002), indicator under certain condition (water temperature ≤ 12 °C) in parentheses; USA: USCODE-2018-TITLE 33 (Chap 26)—Navigation and Navigable Waters; EU: Council Directive (91/271/EEC); Japan: General Standard of Drainage (一般排水基準(法) in Japanese), indicator of daily maximum in parentheses; Singapore: Singapore Wastewater Effluent Discharge Standards (see http://www.water-treatment.com.cn/resources/discharge-standards/singapore.htm (accessed on 2 May 2022)); and Malaysia: Environmental Quality (Sewage) Regulations 2009.

## Data Availability

Researchers are required to apply for permission to use the data.

## Supplementary Materials

The following supporting information can be downloaded at: https://www.mdpi.com/article/10.3390/ijerph19127291/s1, Table S1: Number of questionnaires and regional distribution; File S1: Questionnaire on researching personal environmental and ecological perception.
