# Supplementary material for: Does Individuals’ Perception of Wastewater Pollution Decrease Their Self-Rated Health? Evidence from China
Source: Int J Environ Res Public Health. 2022 Jun 14;19(12):7291. doi: 10.3390/ijerph19127291 (PMC9223579; doi:10.3390/ijerph19127291)
Supplement: Supplementary file 1 [file ijerph-19-07291-s001.zip › ijerph-1733804-supplementary.pdf]

Supplementary Materials

Table S1

Number of questionnaires and regional distribution

**Province (Or equivalent)**	**City**	**County**	**Count (County)**	**Count (City)**	**Count (Prov)**	**Total**
**Anhui**	Anqing	Huaining	1	11	264	6112
		Qianshan	1			
		Susong	4			
		Taihu	1			
		Wangjiang	4			
	Bengbu	Bengshan	9	38		
		Guzhen	2			
		Huaiyuan	2			
		Huaishang	2			
		Longzihu	21			
		Wuhe	1			
		Yuhui	1			
	Bozhou	Lixin	9	19		
		Mengcheng	1			
		Qiaocheng	5			
		Guoyang	4			
	Chizhou	Dongzhi	2	4		
		Guichi	2			
	Chuzhou	Dingyuan	1	25		
		Fengyang	15			
		Lai'an	1			
		Langya	1			
		Mingguang	2			
		Nanqiao	1			
		Quanjiao	2			
		Tianchang	1			
		-	1			
	Fuyang	Linquan	18	35		
		Yingzhou	4			
		Jieshou	3			
		Yingshang	2			
		Yingquan	2			
		Funan	5			
		Yingdong	1			
	Hefei	Luyang	6	44		
		Yaohai	10			
		Baohe	5			

		Feidong	3		
		Shushan	4		
		Lujiang	7		
		Feixi	6		
		Chaohu	2		
		Changfeng	1		
	Huaibei	Xiangshan	2	6	
		Suixi	4		
	Huainan	Fengtai	4	7	
		Bagongshan	1		
		Tianjiaan	1		
		Datong	1		
	Huangshan	Shexian	2	8	
		Huangshan	1		
		Xiuning	3		
		Tunxi	2		
	Lu'an	Yeji	3	19	
		Jinan	4		
		Jinzhai	1		
		Yu'an	3		
		Huoqiu	2		
		Huoshan	3		
		Shucheng	2		
		-	1		
	Ma'anshan	Yushan	1	5	
		Huashan	2		
		Hexian	1		
		Dangtu	1		
	Suzhou	Yongqiao	6	18	
		Xiao	4		
		Lingbi	5		
		Dangshan	3		
	Tongling	Yi'an	1	6	
		Tongguan	2		
		Zongyang	3		
	Wuhu	Jinghu	1	12	
		Yijiang	2		
		Wuwei	7		
		Nanling	2		
	Xuancheng	Ningguo	1	7	
		Langxi	3		
		Xuanzhou	2		
		-	1		
**Beijing**	Beijing	Fangshan	2	224	224

		Xicheng	4		
		Chaoyang	2		
		Haidian	7		
		Tongzhou	2		
		Shijingshan	1		
		Dongcheng	2		
		Miyun	1		
		-	203		
**Fujian**	Fuzhou	Luoyuan	1	229	447
		Fuqing	227		
		-	1		
	Nanping	Jian'ou	1	5	
		Shunchang	1		
		Yanping	1		
		Songxi	1		
		Shaowu	1		
	Ningde	Xiapu	1	3	
		Fu'an	1		
		Fuding	1		
	Putian	Xianyou	2	2	
	Quanzhou	Yongchun	1	1	
	Sanming	Mingxi	1	2	
		Qingliu	1		
	Xiamen	Tong'an	1	2	
		-	1		
	Zhangzhou	Changtai	200	203	
		Longhai	1		
		-	2		
**Gansu**	Qingyang	Huan	208	208	230
	Zhangye	Minle	1	1	
	Dingxi	Weiyuan	1	1	
	Gannan	Zhuoni	1	2	
		Lintan	1		
	Tianshui	Qingshui	1	1	
	Baiyin	Huining	2	4	
		-	2		
	Lanzhou	-	7	7	
	Linxia	Linxia	1	2	
		Guanghe	1		
	Pingliang	Kongtong	1	3	
		-	2		
	Wuwei	-	1	1	
**Guangdong**	Chaozhou	Raoping	2	2	233

	Dongguan	-	11	11	
	Foshan	Shunde	9	10	
		Nanhai	1		
	Guangzhou	Baiyun	2	28	
		Huangpu	6		
		Panyu	6		
		Tianhe	7		
		Huadu	1		
		Zengcheng	1		
		Liwan	3		
		Haizhu	1		
		Yuexiu	1		
	Heyuan	Longchuan	1	1	
	Huizhou	Huiyang	106	121	
		Huidong	4		
		Huicheng	11		
	Jieyang	Jiedong	2	6	
		Huilai	2		
		Puning	1		
		-	1		
	Meizhou	Wuhua	2	4	
		Meijiang	1		
		Jiaoling	1		
	Qingyuan	Yingde	3	3	
	Shantou	Jinping	1	2	
		Longhu	1		
	Shanwei	Lufeng	1	1	
	Shaoguan	Zhenjiang	1	1	
	Shenzhen	Longhua	1	26	
		Luohu	1		
		Longgang	9		
		Futian	6		
		Baoan	2		
		Pingshan	3		
		Nanshan	3		
		-	1		
	Yangjiang	Yangxi	1	15	
		Jiangcheng	14		
	Zhongshan	-	1	1	
	Zhuhai	Xiangzhou	1	1	
**Guangxi**	Baise	Jingxi	1	15	255
		Pingguo	3		
		Xilin	5		

		Longlin	1		
		Jingxi	2		
		Lingyun	1		
		-	2		
	Guigang	Guiping	1	1	
	Guilin	Resource	1	13	
		Xing an	1		
		Pingle	6		
		-	5		
	Hechi	Luocheng	1	212	
		Du'an	1		
		Huanjiang	6		
		-	204		
	Laibin	Xiangzhou	1	1	
	Liuzhou	Rong'an	1	1	
	Nanning	Qingxiu	1	10	
		Xixiangtang	1		
		Binyang	1		
		Wuming	1		
		-	6		
	-	-	2	2	
**Guizhou**	Anshun	Zhenning	3	331	507
		Ziyun	1		
		-	327		
	Bijie	Weining	140	141	
		Qianxi	1		
	Guiyang	-	3	3	
	Liupanshui	Liuzhi	1	14	
		Panzhou	2		
		-	11		
	Qiandongnan	Kaili	1	2	
		-	1		
	Qiannan	Fuquan	1	4	
		Dushan	2		
		-	1		
	Qianxinan	Xingren	2	4	
		Pu'an	1		
		Xingyi	1		
	Tongren	Jiangkou	1	2	
		Yanhe	1		
	Zunyi	Wuchuan	1	3	
		Chishui	1		
		-	1		

	-	-	3	3	
**Hainan**	Baisha	-	10	10	65
	Changjiang	-	2	2	
	Chengmai	-	4	4	
	Danzhou	-	3	3	
	Dongfang	-	1	1	
	Haikou	Longhua	9	31	
		Meilan	8		
		Qiongshan	7		
		Xiuying	7		
	Lingshui	-	2	2	
	Qionghai	-	3	3	
	Sanya	Tianya	1	4	
		Jiyang	2		
		Haitang	1		
	Wanning	-	1	1	
	Wenchang	-	3	3	
	-	-	1	1	
**Hebei**	Baoding	Yi	3	8	241
		Quyang	1		
		-	4		
	Cangzhou	Hejian	2	2	
	Chengde	Luanping	1	1	
	Handan	Qiu	1	4	
		Cheng'an	2		
		Hanshan	1		
	Hengshui	Taocheng	1	4	
		Shenzhou	1		
		-	2		
	Langfang	Sanhe	1	2	
	Qinhuangdao	Funing	1		
	Shijiazhuang	Zhengding	1	3	
		Xinhua	1		
		-	1		
	Tangshan	Kaiping	1	3	
		-	2		
	Xingtai	Pingxiang	212	213	
		-	1		
	Zhangjiakou	-	1	1	
**Henan**	Hebi	Qi	1	2	74
		Qibin	1		
	Jiyuan	-	1	1	

	Jiaozuo	Xiuwu	1	3	
		Jiefang	1		
		-	1		
	Kaifeng	Tongxu	1	4	
		Longting	2		
		-	1		
	Luoyang	Yiyang	1	2	
		Luolong	1		
	Luohe	Zhaoling	1	1	
	Nanyang	Xixia	1	5	
		Nanzhao	1		
		Xinye	2		
		Wancheng	1		
	Pingdingshan	Ye	1	1	
	Shangqiu	Yongcheng	1	3	
		Minquan	1		
		Liangyuan	1		
	Xinxiang	Xinxiang	1	2	
		Fengqiu	1		
	Xinyang	Xin	1	28	
		Shangcheng	2		
		Shihe	2		
		Gushi	23		
	Zhengzhou	Zhongyuan	5	17	
		Erqi	3		
		Jinshui	8		
		-	1		
	Zhoukou	Huaiyang	1	3	
		Chuanhui	1		
		-	1		
	Zhumadian	Pingyu	1	1	
	-	-	1	1	
**Heilongjiang**	Daqing	-	2	2	330
	Harbin	Shuangcheng	1	47	
		Daowai	1		
		Acheng	1		
		-	44		
	Hegang	Nanshan	1	206	
		-	205		
	Jiamusi	-	71	71	
	Mudanjiang	Ning'an	1	2	
		-	1		
	Qiqihar	Gannan	1	1	

	Suihua	Lanxi	1	1	
**Hubei**	Enshi	Badong	3	14	71
		Xuanen	4		
		Enshi	2		
		Jianshi	2		
		-	3		
	Huanggang	Huangzhou	1	3	
		Wuxue	2		
	Lichuan	-	1	1	
	Shiyan	Danjiangkou	7	7	
	Suizhou	Zengdu	12	24	
		Sui	12		
	Tianmen	-	1	1	
	Wuhan	Wuchang	1	12	
		Huangpi	4		
		Hongshan	6		
		Jiangxia	1		
	Xianning	Tongcheng	2	3	
		-	1		
	Xiangyang	Zaoyang	1	2	
		-	1		
	Yichang	Dianjun	1	2	
		-	1		
	-	-	2	2	
**Hunan**	Changde	Shimen	222	225	246
		-	3		
	Chenzhou	Zixing	1	2	
		Yizhang	1		
	Loudi	Louxing	1	1	
	Shaoyang	Chengbu	1	3	
		Longhui	1		
		Shaodong	1		
	Xiangxi	Baojing	1	2	
		Yongshun	1		
	Yongzhou	Jianghua	1	2	
		Ningyuan	1		
	Zhangjiajie	Cili	1	3	
		-	2		
	Changsha	Yuelu	1	6	
		Ningxiang	1		
		Hengshan	1		
		-	3		
	-	-	2	3	

**Jilin**	Baicheng	-	2	2	70
	Changchun	Chaoyang	1	3	
		Nong'an	1		
		-	1		
	Dunhua	-	1	1	
	Siping	Lishu	5	55	
		Yitong Manchu Autonomous	2		
		Tiexi	3		
		Tiedong	34		
		-	11		
	Songyuan	Ningjiang	1	3	
		Changling	1		
		-	1		
	Yanbian	Yanji	2	3	
		Hunchun	1		
	Liaoyuan	Dongliao	1	1	
	-	-	2	2	
**Jiangsu**	Changzhou	Wujin	9	32	134
		Tianning	9		
		Zhonglou	4		
		Xinbei	8		
		Jintan	1		
		Liyang	1		
	Huai'an	Huaiyin	1	2	
		Lianshui	1		
	Lianyungang	Haizhou	2	3	
		Lianyun	1		
	Nanjing	Qixia	12	29	
		Liuhe	1		
		Gulou	5		
		Jiangning	4		
		Xuanwu	4		
		-	3		
	Nantong	Qidong	1	3	
		Rugao	1		
		Rudong	1		
	Suzhou	Wujiang	1	29	
		Wuzhong	9		
		Gusu	13		
		Xiangcheng	2		
		Zhangjiagang	1		
		Huqiu	3		

	Taizhou	Taixing	1	1	
	Wuxi	Binhu	11	24	
		Liangxi	4		
		Xinwu	5		
		Huishan	3		
		Xishan	1		
	Xuzhou	Suining	1	1	
	Yancheng	Dongtai	1	1	
	Zhenjiang	Yangzhong	6	9	
		Runzhou	2		
		-	1		
**Jiangxi**	Ganzhou	Xunwu	1	2	43
		Zhanggong	1		
	Ji'an	Suichuan	1	15	
		Xingan	1		
		Ji'an	11		
		Jinggangshan	1		
		Yongxin	1		
	Jingdezhen	Zhushan	3	5	
		Fuliang	1		
		Changjiang	1		
	Jiujiang	-	1	1	
	Nanchang	Qingyunpu	2	17	
		Donghu	5		
		Xinjian	5		
		Qingshanhu	3		
		Anyi	1		
		-	1		
	Yichun	Gao'an	1	1	
	Yingtan	Yujiang	1	1	
	Shangrao	-	1	1	
**Liaoning**	Chaoyang	Jianping	3	3	69
	Dalian	Zhongshan	3	47	
		Ganjingzi	18		
		Xigang	3		
		Jinzhou	8		
		Zhuanghe	4		
		Shahekou	6		
		Wafangdian	2		
		Pulandian	2		
		-	1		
	Dandong	Zhen'an	1	4	
		Zhenxin	1		

		Donggang	1		
		-	1		
	Fushun	-	1	1	
	Fuxin	Qinghemen	1	1	
	Huludao	Suizhong	1	1	
	Shenyang	Liaozhong	3	10	
		Huanggu	1		
		Tiexi	2		
		Faku	1		
		Hunnan	1		
		-	2		
	Tieling	Yinzhou	1	1	
	Yingkou	Gaizhou	1	1	
**Inner Mongolia**	Bayan Nur	Urad Front Banner	1	3	231
		-	2		
	Baotou	-	2	2	
	Chifeng	Balin Right Banner	1	14	
		Ningcheng	1		
		Aohan Banner	2		
		Wudan	1		
		Songshan	1		
		-	8		
	Hohhot	Tokto	1	3	
		-	2		
	Hulun Buir	Xin Barag Right Banner	1	3	
		Zhalantun	1		
		-	1		
	Tongliao	Kulun Banner	1	4	
		Jarud Banner	1		
		-	2		
	Ulanqab	Shangdu	2	4	
		Liangcheng	1		
		-	1		
	Xilingol League	Abaga Banner	1	137	
		East Ujimqin banner	5		
		Duolun	6		
		Erenhot	2		
		Sonid Right Banner	25		
		Sonid Left Banner	1		
		Taibus Banner	8		
		West Ujimqin banner	2		
		Xilinhot	52		

		Boarder Yellow Banner	4		
		Zhenglan Banner	14		
		Zhengxiangbai Banner	4		
		-	13		
	Hinggan League	Ulanhot	1	1	
	-	-	60	60	
**Ningxia**	Guyuan	Pengyang	1	5	237
		-	4		
	Shizuishan	Dawukou	3	3	
	Wuzhong	Tongxin	2	4	
		-	2		
	Yinchuan	Xingqing	1	222	
		-	221		
	Zhongwei	Shapotou	1	2	
		-	1		
	-	-	1	1	
**Qinghai**	Haidong	Huzhu	1	125	214
		Minhe	4		
		Ping'an	2		
		Hualong	118		
	Haibei	Menyuan	6	7	
		Qilian	1		
	Haixi	Golmud	2	3	
		Delhi city	1		
	Huangnan	Jianzha	3	3	
	Xining	Chengbei	3	75	
		Chengdong	18		
		Chengxi	12		
		Urban	11		
		Datong	11		
		Huangzhong	12		
		-	8		
	Yushu	-	1	1	
**Shandong**	Dezhou	Linyi	2	4	328
		-	2		
	Heze	-	1	1	
	Jining	Liangshan	1	3	
		Weishan	1		
		-	1		
	Liaocheng	Linjing	1	1	
	Linyi	Pingyi	1	3	

		-	2		
	Qingdao	Licang	1	3	
		Shinan	1		
		-	1		
	Rizhao	Wulian	1	2	
		Donggang	1		
	Weihai	Rongcheng	2	305	
		-	303		
	Weifang	Qingzhou	1	2	
		Anqiu	1		
	Yantai	Longkou	1	4	
		-	3		
**Shanxi**	Datong	Lingqiu	2	2	224
	Jincheng	Zezhou	1	1	
	Linfen	Yaodu	1	1	
	Luliang	Wenshui	1	1	
	Taiyuan	Xiaodian	1	2	
		Wanbailin	1		
	Xinzhou	Hequ	213	215	
		Dingxiang	1		
		-	1		
	Yuncheng	Hejin	1	1	
	-	-	1	1	
**Shaanxi**	Ankang	Hanbin	2	4	43
		Baihe	2		
	Baoji	Fufeng	1	4	
		Fengxiang	1		
		-	2		
	Shangluo	Shanyang	1	3	
		-	2		
	Weinan	Linwei	1	1	
	Xi'an	Yanta	12	27	
		Lantian	1		
		Lianhu	2		
		Weiyang	4		
		Beilin	2		
		Chang'an	3		
		Baqiao	1		
		Xincheng	1		
		-	1		
	Xianyang	Yangling	1	1	
	Yan'an	Baota	2	2	
	Yulin	Fugu	1	1	

**Shanghai**	Shanghai	Baoshan	1	50	50
		Fengxian	1		
		Hongkou	2		
		Huangpu	1		
		Jiading	2		
		Minhang	4		
		Pudong	23		
		Putuo	2		
		Qingpu	2		
		Songjiang	2		
		Xuhui	1		
		Yangpu	5		
		Changning	4		
**Sichuan**	Ngawa	Barkam	1	1	537
	Chengdu	Shuangliu	6	12	
		Xindu	1		
		Jinjiang	1		
		Chongzhou	1		
		-	3		
	Dazhou	Dachuan	3	288	
		Quxian	12		
		Xuanhan	268		
		Wanyuan	1		
		Dazhu	2		
		-	2		
	Garzê	Kangding	1	3	
		Danba	1		
		-	1		
	Guangyuan	Jiange	1	1	
	Liangshan	Yanyuan	1	220	
		Ningnan	1		
		Yanyuan	218		
	Meishan	Renshou	1	1	
	Mianyang	Fucheng	1	2	
		Santai	1		
	Nanchong	Nanbu	2	2	
	Panzhihua	Western	1	1	
	Yibin	Changning	1	4	
		Junlian	1		
		-	2		
	Ziyang	Anyue	1	1	
	-	-	1	1	
**Tianjin**	Tianjin	Baodi	1	40	40

		Beichen	6		
		Heping	3		
		Hebei	1		
		Hedong	7		
		Hexi	5		
		Jizhou	2		
		Jinghai	3		
		Jinnan	1		
		Xiqing	1		
		Ninghe	3		
		Nankai	6		
		-	1		
**Tibet**	Changdu	Chagyab	21	21	103
	Lhasa	-	26	26	
	Nagqu	Shenzha	1	55	
		Biru	1		
		-	53		
	Shannan	Nagarzê	1	1	
**Xinjiang**	Aksu	Awat	1	243	355
		-	242		
	Karamay	Dushanzi	2	3	
		-	1		
	Urumqi	-	3	3	
	Ili	He Cheng	103	103	
	-	-	3	3	
**Yunnan**	Baoshan	Tengchong	1	2	45
		-	1		
	Chuxiong	Nanhua	1	1	
	Dali	Eryuan	2	6	
		Yongping	1		
		Heqing	1		
		-	2		
	Dehong	Lianghe	1	1	
	Diqing	Shangri-La	4	4	
	Honghe	Shiping	2	5	
		Mengzi	1		
		-	2		
	Kunming	Anning	1	7	
		Xishan	1		
		-	5		
	Lincang	Fengqing	1	2	
		-	1		
	Pu'er	-	1	1	

	Qujing	Fuyuan	1	4	
		Xuanwei	1		
		-	2		
	Wenshan	Qiubei	7	9	
		Yanshan	1		
		Wenshan	1		
	Zhaotong	Zhaoyang	1	3	
		Yongshan	1		
		Yiliang	1		
**Zhejiang**	Hangzhou	Riverside	2	90	129
		Gongshu	1		
		Shangcheng	1		
		Jianggan	5		
		Tonglu	63		
		Xihu	5		
		Xiacheng	3		
		Xiaoshan	2		
		Yuhang	4		
		-	4		
	Jiaxing	Xiuzhou	1	7	
		Haining	3		
		Haiyan	2		
		Tongxiang	1		
	Jinhua	Pan'an	1	2	
		-	1		
	Ningbo	Yuyao	1	6	
		Cixi	1		
		Jiangbei	1		
		Zhenhai	1		
		Haishu	1		
		-	1		
	Quzhou	Jiangshan	7	8	
		Kecheng	1		
	Shaoxing	Yuecheng	1	12	
		Zhuji	10		
		-	1		
	Taizhou	Tiantai	1	2	
		Huangyan	1		
	Wenzhou	Ruian	1	2	
		Yueqing	1		
**Chongqing**	Chongqing	Beibei	1	73	73
		Dianjiang	1		
		Dazu	1		

	Hechuan	1	
	Fengjie	3	
	Jiangbei	1	
	Kaizhou	1	
	Pengshui	3	
	Nan'an	12	
	Qijiang	1	
	Qianjiang	7	
	Shizhu	1	
	Shapingba	3	
	Tongnan	1	
	Wanzhou	15	
	Xiushan	4	
	Yongchuan	1	
	Youyang	4	
	Yubei	5	
	Yuzhong	2	
	Yunyang	1	
	-	4	

Note: “-” denotes that the respondents refused to disclose their location.

Supplementary File S1

Questionnaire on researching personal environmental and ecological perception

Investigator: Research Date:

(Note: Respondents need to answer based on their own local actual conditions and their own perceptions. The investigators ensure that the respondent's sensitive information will be kept strictly confidential)

Part A Basic information of the respondents

1. Gender: 0. Male; 1. Female;

2. Age: 1. Under 30 years old; 2. Between 31-50 years old; 3. Above 50 years old

3. Nationality: _______

4. Education level: 1. Primary school or below 2. Middle school 3. High school 4. College or university 5. Master degree or above;

5. Family size: _____ persons;

6. Type of household registration: 0. Agricultural Hukou; 1. Non-agricultural Hukou;

7. Address: __________ Town (township) _________ Village, __________ County, __________ City, __________ Province

Part B Respondents' overall views on environmental pollution

1. Do you think there is current local environmental pollution?

A. Yes, serious; B. Yes, not too serious; C. No; D. Don't know

2. What do you think the current local environment has changed compared to 5 years ago?

A. Much better; B. A little better; C. Fair; D. A little worse; E. A lot worse

3. Same as question 2, how has it changed compared to 10 years ago?

A. Much better; B. A little better; C. Fair; D. A little worse; E. A lot worse

4. What do you think is the main source of local solid pollution? (Multiple choice)

A. Industrial solid waste; B. Agricultural solid waste; C. Medical solid waste; D. Domestic waste;

E. Other; F. Don’t know

5. What is the main type of wastewater pollution locally? (Multiple choice)

A. Industrial wastewater; B. Agricultural wastewater; C. Domestic wastewater; D. Others; E. Don’t know

6. Do you think there is ecological or environmental damage at present?

A. Yes, serious; B. Yes, not too serious; C. No; D. Don’t know

7. Do you think the current environment has an impact on your life?

A. Yes, serious; B. Yes, not serious; C. No effect; D. It doesn’t matter

8. Submission question 7, if there is an impact, which aspects of you have been mainly affected? (Multiple choice)

A. Willingness to buy a house; B. Work; C. Mood; D. Leisure; E. Health; F. Others (specify) _____

9. What do you think the current local government’s environmental protection is doing?

A. Very good; B. Relatively good; C. Average; D. Not very good; E. Very poor; F. No protection at all; G. Don’t know

10. What do you think the local environment will be like in the next three years?

A. It has become much better than it is now; B. It has become better than it is now; C. There would be no change; D. It would become a little worse than present; E. It would become really worse than present; F. Don’t know

11. How confident are you that local environmental conditions will improve?

A. Very confident; B. Relatively confident; C. Average; D. Not so confident; E. Not confident at all

12. Do you think the local government will increase investment in environmental governance in the future?

A. Yes; B. No; C. Don’t know

13. Where do you think the government should start to manage the environment first?

A. Residents’ living environment; B. Regional ecological environment; C. Don’t know

14. What do you think of the current local comprehensive environmental situation?

A. Very good; B. Relatively good; C. Average; D. Not very good; E. Very poor

Part C Respondents' specific understanding of the environment（Ellipsis）

Part D Respondents' perceptions of happiness and health

1. How happy do you think your life is?

A. Very happy; B. Relatively happy; C. Average; D. Not very happy; E. Very unhappy

2. What do you think of your health status at present?

A. Very healthy; B. Relatively healthy; C. Average; D. Not very healthy; E. Very unhealthy

3. Compared to the last year, how do you evaluate your current health status?

A. Better; B. No change; C. Worse

4. Compared to your peers, how do you evaluate your current health status?

A. Better than their peers; B. About the same; C. Worse than their peers

5-7 (**Ellipsis**)

8. Do you think the morning of the day is the best?

A. Always; B. Often; C. Sometimes; D. Occasionally; E. Never

9. Do you cry or feel like crying?

A. Always; B. Often; C. Sometimes; D. Occasionally; E. Never

10. Do you often feel sullen and depressed?

A. Always; B. Often; C. Sometimes; D. Occasionally; E. Never

11. Do you often have trouble sleeping?

A. Always; B. Often; C. Sometimes; D. Occasionally; E. Never

12. Are you fatigued with no reason?

A. Always; B. Often; C. Sometimes; D. Occasionally; E. Never

13. Do you find it easy to do things you often do?

A. Always; B. Often; C. Sometimes; D. Occasionally; E. Never

14. Are you feeling upset and find it difficult to calm down?

A. Always; B. Often; C. Sometimes; D. Occasionally; E. Never

15. Do you find it easy to make a decision?

A. Always; B. Often; C. Sometimes; D. Occasionally; E. Never

16. You feel like a useful person, does anyone need you?

A. Always; B. Often; C. Sometimes; D. Occasionally; E. Never

17. Do you think your life is very interesting?

A. Always; B. Often; C. Sometimes; D. Occasionally; E. Never

Note: Due to the request of financial founders, we could only release several parts of questions (or some questions in a part) that involves in our research, namely, the Question Part of respondents' socioeconomic information, the individual's views on environmental pollution, and the individual evaluation of health status and subjective wellbeing.
